# Vision

**DOI:** 10.3390/vision1010001

**Published:** 2017-02-20

**Authors:** Andrew Parker

**Affiliations:** Department of Physiology, Anatomy and Genetics, University of Oxford, Oxford OX1 3PT, UK; andrew.parker@dpag.ox.ac.uk; Tel.: +44 1865 272504

*Vision* is a new open-access journal covering all aspects of experimental vision research and clinical science. We particularly want to create a forum for the development of a dialogue between fundamental laboratory-based research and clinical research practice.

We are not aligned to any particular discipline or professional training. Thus we welcome and encourage research contributions and reviews that are rooted in any of the following: anatomy, cell biology, physiology, genetics, psychology, engineering, computational science, ophthalmology, neurology, optometry, clinical medicine and other disciplines. The uniting feature is that this journal will cover all aspects of human and biological vision and how its normal functioning may go wrong.

An important question is whether another journal in the field of vision is needed. Vision research has developed tremendously over the last years and has been well served by some of the available journals. We feel that a new journal, with no historical or societal affiliations, offers a chance for a broad interdisciplinary coverage and a means of highlighting the increasing number of interesting results and ideas arising from this convergence. We are therefore convinced that the launch of a truly interdisciplinary journal in the area of vision science is worthwhile, timely and necessary. *Vision* is a journal for, and by, all vision scientists, committed to the advancement of their field of interest.

Behind the launch of a new journal lies a fundamental question about why we do research at all. Our answers to this are thoroughly conventional. We want to increase understanding and fundamental knowledge about how we see. We want to see this knowledge applied to the benefit of people. This can happen individually when people receive clinical treatment. But benefit can also extend to economic and social goods, when new knowledge stimulates the creation of new systems or devices, or new ways of conducting commerce or business.

For these broader reasons, we are committed to the open-access model of publication. We feel that knowledge generated by the use of public or charitable funds should be available to all. We think that this is an important step in demonstrating and providing public benefit from the use of those funds. There will be readers who have concerns about the open-access model. It is argued that the model encourages the publication of anything, even material of weak quality, provided the author can pay. The editors are jointly committed to advancing high standards to prevent any perceived slide in this direction.

We feel fortunate in the association with MDPI. The publishing house is entirely devoted to open-access publishing. The standard costs of publication are modest and, until the our new journal acquires an impact factor rating, the cost of publication will be zero as part of the launch program for the new *Vision* journal. When I visited the journal offices in Wuhan (PR China) during the summer of 2016, I found an in-house staff who are knowledgeable in biomedical science, young, enthusiastic and keen to support the greater goal of improving and disseminating scientific knowledge.

MDPI have demonstrated their commitment to open access by concluding a data archiving arrangement with the Swiss National Library System. All papers published in *Vision* (and other MDPI journals) are digitally archived within this publicly available system. Thus, in the event of a major default by the journal publisher or editors or other archiving sources, there is a guarantee that the work of contributors will continue to be available through the Swiss Government’s library system. (For example, other titles in the MDPI list of journals can be currently accessed on-line through *e-Helvetica Access.*) This seems to be a useful way of bringing the public and private systems together to the greater benefit of both.

Whether or not articles are cited is another line of defence against critics of the “pay-to-publish” model. The limitations of citation indices as a model of research quality have been widely debated, but one of the less contentious uses of volume of citations is in constructing the impact factor of journals. It is well understood that the largest impact factors are associated with journals with a very broad coverage, so there is a limit to growth for a journal such as we launch here. Nonetheless, we recognize that as a new journal we are at the bottom of a ladder, which we greatly aspire to climb.

We also want to participate in efforts to build on the progress achieved through the concept of open-access to the wider goal of open science. The editorial board is considering a number of options, including on-line presentation of primary data, reviewer recognition, possible use of open reviewing, or even a section of the journal with no reviewing at all. All these policy shifts have their cautionary notes attached to them, but the opportunity provided by launching a new journal is that we can afford to take some risks and see how they work out. The editors fully intend to explore these areas in order to develop the publishing model.

We see the next 10 years as an exciting time for scientific research and publishing. The surge of interest in what science can achieve, especially in highly populated countries such as India and China, is bound to bring a new stream of talent to scientific discovery and practice. Those of us whose first language is English are fortunate that the dominant scientific discourse is carried out in our language. But English has also become the common currency for more informal scientific exchange, in many ways being the most widely dispersed, second or alternative language worldwide.

Our immediate plans are to widen the membership of the editorial board and to encourage and commission the submission of groups of articles with a single theme to make a “Special Issue” of the journal. Our official launch date is March 2017 and we look forward to that time point, when we aim to bring a new and differently-toned voice to the debate around the table in the area of vision research.


Andrew Parker Oxford 11 Feb 2017


